# Neural correlates of the behavioral-autonomic interaction response to potentially threatening stimuli

**DOI:** 10.3389/fnhum.2012.00349

**Published:** 2013-01-16

**Authors:** Tom F. D. Farrow, Naomi K. Johnson, Michael D. Hunter, Anthony T. Barker, Iain D. Wilkinson, Peter W. R. Woodruff

**Affiliations:** ^1^Sheffield Cognition and Neuroimaging Laboratory, Academic Clinical Psychiatry, University of SheffieldSheffield, UK; ^2^Department of Medical Physics, Sheffield Teaching Hospitals NHS TrustSheffield, UK; ^3^Academic Unit of Radiology, University of SheffieldSheffield, UK

**Keywords:** functional magnetic resonance imaging (fMRI), skin conductance response (SCR), emotional salience, autonomic arousal, threat, affective tone

## Abstract

Subjective assessment of emotional valence is typically associated with both brain activity and autonomic arousal. Accurately assessing emotional salience is particularly important when perceiving threat. We sought to characterize the neural correlates of the interaction between behavioral and autonomic responses to potentially threatening visual and auditory stimuli. Twenty-five healthy male subjects underwent fMRI scanning whilst skin conductance responses (SCR) were recorded. One hundred and eighty pictures, sentences, and sounds were assessed as “harmless” or “threatening.” Individuals' stimulus-locked, phasic SCRs and trial-by-trial behavioral assessments were entered as regressors into a flexible factorial design to establish their separate autonomic and behavioral neural correlates, and convolved to examine psycho-autonomic interaction (PAI) effects. Across all stimuli, “threatening,” compared with “harmless” behavioral assessments were associated with mainly frontal and precuneus activation with specific within-modality activations including bilateral parahippocampal gyri (pictures), bilateral anterior cingulate cortex (ACC) and frontal pole (sentences), and right Heschl's gyrus and bilateral temporal gyri (sounds). Across stimulus modalities SCRs were associated with activation of parieto-occipito-thalamic regions, an activation pattern which was largely replicated within-modality. In contrast, PAI analyses revealed modality-specific activations including right fusiform/parahippocampal gyrus (pictures), right insula (sentences), and mid-cingulate gyrus (sounds). Phasic SCR activity was positively correlated with an individual's propensity to assess stimuli as “threatening.” SCRs may modulate cognitive assessments on a “harmless–threatening” dimension, thereby modulating affective tone and hence behavior.

## Introduction

Constantly changing environmental stimuli are rapidly processed by the brain to allow reorienting of cognitive resources such as attention toward possible threats (Öhman et al., 2001a,b). Such potential threats are identified by their emotional salience—a stimulus's state or quality of standing out relative to neighboring stimuli. One output of this stimulus-relevance cognitive processing is via the autonomic nervous system (ANS), controlling visceral functions such as perspiration, heart rate, respiration, and pupil diameter. However, due to positive and negative feedback between the cognitive and autonomic systems (Hugdahl, 1996), cognitive processing may be modulated by state or trait ANS activity, thereby subtly influencing how we attend to our environment, which in turn affects our behavior. Previous functional MRI studies have examined the BOLD response to threat processing, but mostly without measuring the ANS component.

Affective tone, an “emotional coloring” of the mental state accompanying every act or thought, arises from a dynamic interaction between cognitive assessment and ANS activity (Ross, 1997). Disturbance of this dynamic interaction, for example, in schizophrenia, may manifest as “sinister attribution bias” in which patients attribute negative connotations to apparently benign situations (Peer et al., 2004; Premkumar et al., 2008; Cohen and Minor, 2010). Physiological parameters such as ANS arousal which underpin affective tone are likely to vary along continua, both within healthy individuals and within pathological states (Wout et al., 2004; Horan et al., 2008; van Os et al., 2009). Hence, individuals within the “healthy” continuum may demonstrate varying levels of ANS and BOLD activity in response to potentially threatening stimuli which could influence the way in which they perceive stimuli and thereby interpret the world (Martin and Penn, 2001; Allen et al., 2007).

Emotionally salient pictures have been reported to activate amygdala, ventromedial prefrontal cortex (vmPFC), posterior hippocampus, and visual cortex (Kesler-West et al., 2001; Öhman et al., 2001b; Vuilleumier et al., 2001; Anders et al., 2004; Lee et al., 2004; Northoff et al., 2004; Heinzel et al., 2005; Garrett and Maddock, 2006; Grimm et al., 2006; Bryant et al., 2008; Premkumar et al., 2008; Kemp et al., 2009). However, many of these studies have used threatening (i.e., angry or fearful) faces. Faces, irrespective of emotion displayed, have specialized brain regions associated with their perception (Kesler-West et al., 2001; Narumoto et al., 2001; Vuilleumier et al., 2001; Britton et al., 2006; Tsao et al., 2006; Tsao and Livingston, 2008) and are restricted in how the displayed emotion is interpreted by healthy individuals (Calder et al., 2001) and should therefore be viewed as a “special case” of threat perception rather than a general exemplar (Britton et al., 2006). In contrast, many non-face stimuli could be described as “threat-ambiguous” in that they are open to subjective interpretation, based on previous experience, personality traits (Gard and Kring, 2009), and state levels of cognitive and autonomic arousal (VaezMousavi et al., 2007; Coccaro et al., 2009). Hence, for the “picture” condition in the current study, we used non-face stimuli. Furthermore, all stimuli (pictures, sentences, and sounds) were piloted to ensure that many were not at the extreme ends of a “harmless-threatening” continuum. This allowed us to analyze behavioral responses on an individual basis (rather than pre-categorizing stimuli at the beginning of the study as either “harmless” or “threatening”).

Studies of visually presented threat-related words have reported activation of the left inferior frontal gyrus (IFG) (Blackwood et al., 2000) and amygdala (together with left lingual gyrus and posterior parahippocampal gyrus; Isenberg et al., 1999; Compton et al., 2003). Previous research into the neural bases of pleasant and unpleasant sounds has mainly concerned music (Blood et al., 1999; Koelsch et al., 2005; Pallesen et al., 2005; Eldar et al., 2007). In their PET study, Blood et al. (1999) reported rCBF changes in paralimbic and neocortical areas when musical consonance and dissonance were varied (synonymous with a pleasant to unpleasant range). Notably, these neocortical areas were distinct from areas of primary auditory cortex (involved in pitch and loudness discrimination) or secondary auditory cortex (involved in harmonic, melodic, and rhythmic pattern detection). The relative lack of neuroimaging research into auditory compared with visual stimuli probably has much to do with the difficulties of presenting sounds in a noisy MRI scanner (Di Salle et al., 2003). In the present study, we minimized the difficulties associated with auditory interference by using a “sparse” EPI protocol which allows stimuli to be presented during silent gaps in the scanner sequence.

Previous research on threat perception has also examined response times (RTs) to threatening or negatively valenced stimuli (Cloitre et al., 1992; Estes and Verges, 2008), and counter-intuitively reported *increased* RTs to threatening compared with neutral stimuli. One possible explanation for this finding is that salient stimuli produce opposing effects on attention and behavior such that salience facilitates the identification of threat but slows or inhibits responses to it (Estes and Verges, 2008).

In summary, a number of previous researchers have investigated neural responses to emotionally salient visual and auditory stimuli, though these studies have often involved the “special case” of faces or unambiguous stimuli which were pre-categorized as positive or negative (or “harmless” or “threatening” or “pleasant” or “unpleasant”). By recording SCRs and fMRI BOLD signal to individually rated stimuli we sought to investigate the modulating effect of ANS arousal on brain activation. This concurrent collection of fMRI and SCR data allowed us to examine what we term a psycho-autonomic interaction effect [PAI; comparable with the more often reported psychophysiological interaction effects (PPI)] to “threat-ambiguous” stimuli. Specifically, this convolution methodology allowed examination of BOLD responses attributable to an interaction between autonomic and behavioral responses above and beyond those activations attributable to autonomic and behavioral responses separately. We chose to use pictures, sentences and sounds to allow investigation of stimulus-modality-dependent and -independent factors.

We hypothesized that stimuli subjectively assessed as “threatening” compared with those assessed as “harmless” would be associated with increased RTs (Cloitre et al., 1992; Estes and Verges, 2008) and SCR amplitudes (Hugdahl, 1996). We also hypothesized that stimuli subjectively assessed as “threatening,” irrespective of modality or accompanying phasic SCR, would be associated with increased amygdala activity compared with stimuli assessed as “harmless” (Bishop et al., 2004; Bertolino et al., 2005). We furthermore hypothesized modality-specific activations to “threatening” compared with “harmless” stimuli, specifically, (1) vmPFC, posterior hippocampus, and visual cortex to pictures (Lee et al., 2004; Northoff et al., 2004; Heinzel et al., 2005; Garrett and Maddock, 2006; Grimm et al., 2006); (2) IFG to sentences (Isenberg et al., 1999; Blackwood et al., 2000; Compton et al., 2003); and (3) auditory cortex to sounds (Blood et al., 1999; Koelsch et al., 2005; Pallesen et al., 2005; Eldar et al., 2007). Finally, we hypothesized that phasic SCR activity would be associated with activation of dorso-posterior brain regions (Fredrikson et al., 1998; Patterson et al., 2002) and that PAIs would show dissociable, between-modality activations. In light of the continuum of neuropsychological profiles in healthy volunteer cohorts (Martin and Penn, 2001; Wout et al., 2004; Horan et al., 2008; van Os et al., 2009), we also sought to investigate the influence of schizotypal personality traits on the recorded autonomic and behavioral responses.

## Materials and methods

### Ethics statement

All subjects gave written informed consent. The study was approved by the North Sheffield Research Ethics Committee.

### Stimulus development and piloting

Sixty picture stimuli from the International Affective Picture System (IAPS; Lang et al., 1997) and sixty sentence and sound stimuli developed within our laboratory were piloted on large cohorts (>65) of healthy subject as to whether they were “harmless” or “threatening.” Individual stimuli varied considerably as to the percentage of raters subjectively assessing them as threatening thereby confirming their subjective threat-ambiguous nature. Experimental stimuli used are listed in Appendix Table A1.

### Subjects and neuropsychological assessment

Twenty-five healthy right-handed males (22 ± 2 years old; estimated IQ—National Adult Reading Test, NART; Nelson, 1982 113 ± 6; range 97–123; 16 ± 1 years of education) participated in the study. Study recruitment inclusion criteria comprised being aged 20–35, male, right handed, no current or previous significant neurological or psychiatric disorder, normal or corrected-to-normal vision, no hearing impairment and no general contraindication to MR imaging. Personality-based neuropsychological data were collected from all subjects. Oxford-Liverpool Inventory of Feelings and Experiences sub-scale scores (O-LIFE; Mason et al., 1995; Mason and Claridge, 2006) were: “Unusual Experiences” 3 ± 4, range 0–17 (mean ± SD); “Cognitive Disorganization” 6 ± 5, range 0–17; “Introvertive Anhedonia” 3 ± 2, range 0–6; and “Impulsive Nonconformity” 8 ± 4, range 2–19. Empathy Quotient scale scores (EQ; Baron-Cohen and Wheelwright, 2004) were 46 ± 10; range 32–70; and Paranoia and Suspiciousness Questionnaire scores (PSQ; Rawlings and Freeman, 1996) were 9 ± 6; range 2–27. These tests were chosen to measure individual personality traits, which may be associated with a vulnerability to schizoptypal behavior (psychosis-proneness) and hence a tendency to over-attribute threat (Braunstein-Bercovitz, 2000).

### Intra-scanner SCR recording

ANS activity was measured via skin-conductance response (SCR) recording. A typical phasic SCR is temporally very similar to the BOLD hemodynamic response and is therefore a suitable measure with which to sub-average or convolve fMRI data. MR-compatible SCR equipment was based on a battery powered, electrically isolated, same electrode configuration implementation of a previously published method (Shastri et al., 2001). SCRs sampled at 20 Hz from the medial phalange of the left index and middle fingers, using 8 mm diameter Ag/AgCl electrodes were recorded concurrently with fMRI and behavioral response data.

### fMRI imaging

**Figure 1:**
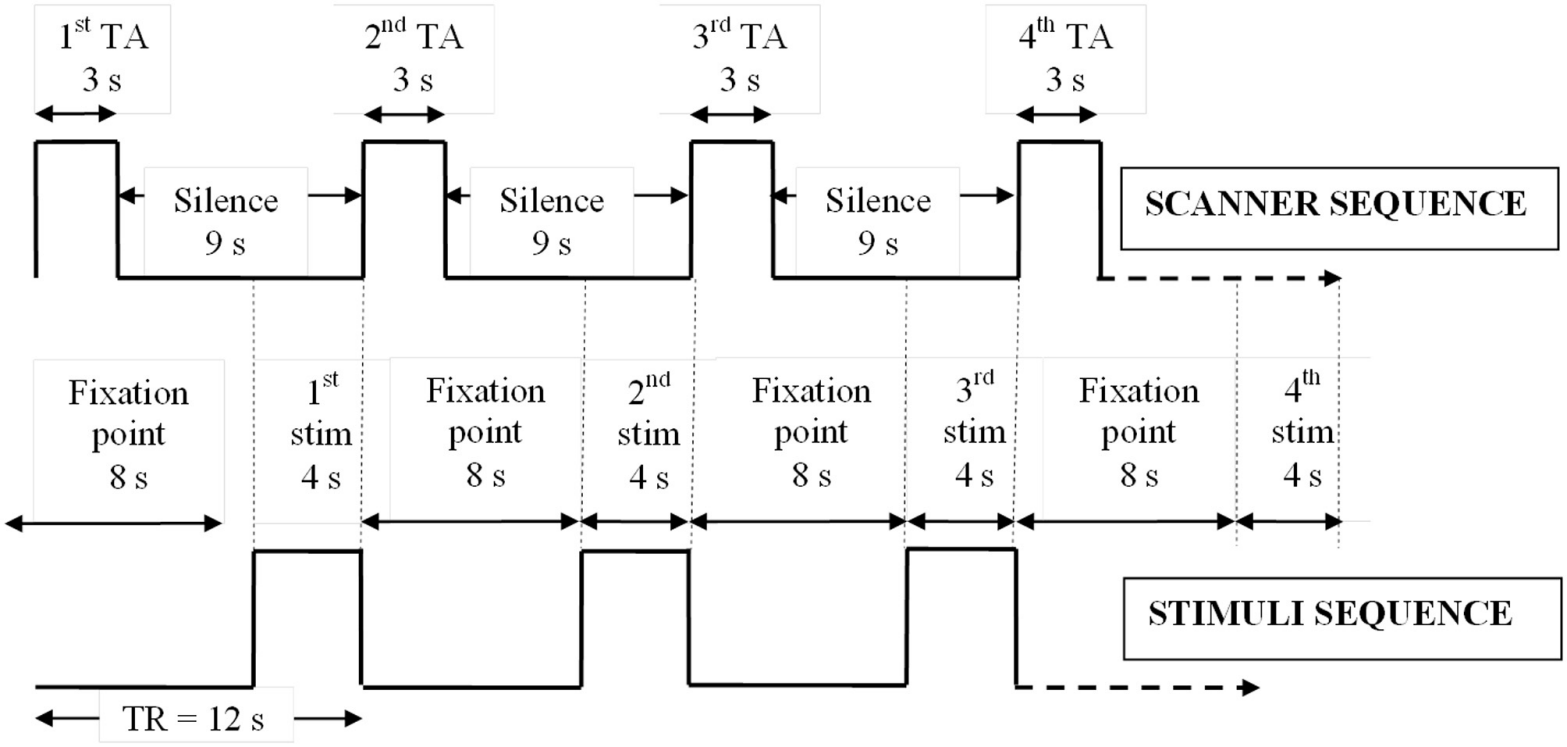
**Sparse EPI protocol.** Relative timings of stimulus presentations and sparse scanner sequence showing how stimuli were delivered in silence immediately prior to fMRI data collection. TA, acquisition time; TR, repetition time; stim, stimulus; s, seconds.

Subjects underwent three 12 min fMRI scans (EPI “sparse” sequence; 60 time points; TR = 12 s; TA = 3 s; TE = 40 ms; SENSE factor = 1.5; FOV = 240 mm; matrix size = 128 × 128, 32 × 4 mm thick contiguous axial slices) at 1.5 Tesla (Eclipse, Philips Medical Systems, Ohio, USA). This data acquisition sequence setup yielded a voxel size of 1.8 × 1.8 × 4 mm. The sparse sequence allows stimuli to be delivered during scanner silent periods (apart from the noise of the helium compressor pump), and for data acquisition to be targeted at a period immediately after task completion, utilizing the physiological delay and dispersion between neuronal activity and its resulting hemodynamic response (Eden et al., 1999). In an order-counterbalanced design, subjects viewed pictures or sentences via a head-coil mounted mirror or listened to sounds via electrostatic headphones. All 180 stimuli (60 pictures, sentences, and sounds; Appendix Table A1) were presented for 4 s each during scanner silence, immediately followed by 3 s of fMRI signal acquisition and a further 5 s of scanner silence (Figure 1). Hence a new stimulus was presented every 12 s (Figure 1). Between presentation of individual pictures and sentences, and continuously during the presentation of sounds, a centrally located fixation cross was displayed. Throughout all scans, the words “Harmless” and “Threatening” were displayed at the bottom of the screen, in a laterality-balanced design (i.e., for half the subjects “Harmless” was displayed on the left of the screen and on the right for the other half of subjects). In a forced-choice design, subjects behaviorally assessed each stimulus as subjectively “harmless” or “threatening” via an intra-scanner button box using their right index and middle fingers.

### SCR data analyses

SCR traces (14,400 data points per 12-min scan) were analyzed in Ledalab v.3.2.9 (www.ledalab.de/; Benedek and Kaernbach, 2010a) using the Continuous Decomposition Analysis method to distinguish the phasic (driver) information from the underlying tonic sudomotor nerve activity. Raw SCR data were smoothed via convolution with a Hann window to reduce error noise and fitted to a bi-exponential Bateman function. Data were optimized by a conjugated gradient descent algorithm to reduce the error between them and the inbuilt SCR model. These processing steps allowed computation of a stimulus-locked “integrated skin conductance response” (ISCR), a time-integration of the continuous phasic activity for each stimulus. The ISCR thus represents an unbiased and time-sensitive measure of sympathetic activity in response to each stimulus (Benedek and Kaernbach, 2010b). For investigating whether SCRs may modulate RTs, each stimulus epoch was also classified via Mindware EDA 2.40 (Mindware Technologies Ltd., OH, USA) as having a significant phasic SCR “present” or “absent” (“a ‘typical’ SCR comprising trough, peak and half-return components, identified within 12 s of stimulus onset; trough-to-peak amplitude = 0.15 μS”). Custom MATLAB scripts (v. R2007b; The MathWorks, Inc., Sherborn, MA, USA) extracted stimulus-locked peak amplitude data for group-averaging of SCRs within and across subjects.

### fMRI data analyses

Functional MRI data were analyzed in SPM8 (Wellcome Department of Imaging Neuroscience, London; www.fil.ion.ucl.ac.uk/spm/) implemented in MATLAB v. R2007b on a PC. The EPI images for each run were corrected for head movement by affine registration using a two-pass procedure by which images were initially realigned to the first image and subsequently to the mean of the realigned images. After realignment, the mean EPI image for each run was spatially normalized to the Montreal Neurological Institute (MNI; Mazziotta et al., 2001) single subject template using the unified segmentation approach (Ashburner and Friston, 2005). The resulting parameters of a discrete cosine transform, which define the deformation field necessary to move the data into the space of the MNI tissue probability maps, were then combined with the deformation field transforming between the latter and the MNI single subject template. The ensuing deformation was applied to the individual EPI volumes, which were thereby transformed into the MNI single-subject space and resampled at 2 × 2× 2 mm voxel size. The normalized images were smoothed using a 6 mm full-width at half-maximum Gaussian kernel to meet the statistical requirements of the General Linear Model and to compensate for residual macro-anatomical variations. For each scan (three per subject), ISCR data (one data point per stimulus epoch) and each individual's harmless/threatening behavioral data were used for regression analysis. At this first level of analysis the BOLD responses were convolved with a canonical hemodynamic response function (HRF), and its temporal derivative. The silent periods of the EPI sequence were modeled in the design matrix by separately specifying the TR (12 s) and TA (3 s). Given the significant differences in reaction times between “harmless” and “threatening” assessments (see “Results” section), an additional reaction time regressor was also added to the model. Hence, for each of the 75 scans, three regression matrices were created: (1) an 8-column regression matrix comprising 1 column of ISCR data, 1 column of reaction time data and 6 columns of subject's movement parameters (obtained from the preprocessing realignment stage); (2) an 8-column regression matrix comprising 1 column of individual behavioral data (harmless = −1; threatening = 1), 1 column of reaction time data and 6 columns of subject's movement parameters; and (3) a 10-column regression matrix comprising 1 column of the convolution between ISCR and behavioral response, 2 columns of separate ISCR and individual behavioral data, 1 column of reaction time data and 6 columns of subject's movement parameters. This final 10-column matrix allowed examination of BOLD responses attributable to an PAI; i.e., brain activity above and beyond those activations separately attributable to the ISCR and behavioral data. These first-level regression analyses were group-averaged at the second-level using a fully flexible factorial design, with factors of subject and modality (picture, sentence, or sound). In this random-effects model, we allowed for violations of sphericity by modeling non-independence across images from the same subject and unequal variances between conditions and subjects as implemented in SPM8. In line with recent guidelines (Lieberman and Cunningham, 2009), analysis of our novel and exploratory complex social neuroscience paradigm was conducted at a significance threshold of *p* < 0.001 uncorrected for multiple comparisons with a minimum extent threshold of 10 voxels. Analysis of the neural correlates of electrodermal activity (i.e., SCR) which has previously been shown to be associated with robust functional activations (Fredrikson et al., 1998; Patterson et al., 2002) was conducted at a significance threshold of *p* < 0.05 corrected for family wise error (FWE). MNI coordinates of all supra-threshold voxels were transformed into Talairach coordinates (Talairach and Tournoux, 1988) using the “mni2tal.m” Matlab script (http://imaging.mrc-cbu.cam.ac.uk/imaging/MniTalairach).

## Results

### Behavioral, physiological, and neuropsychological

**Figure 2:**
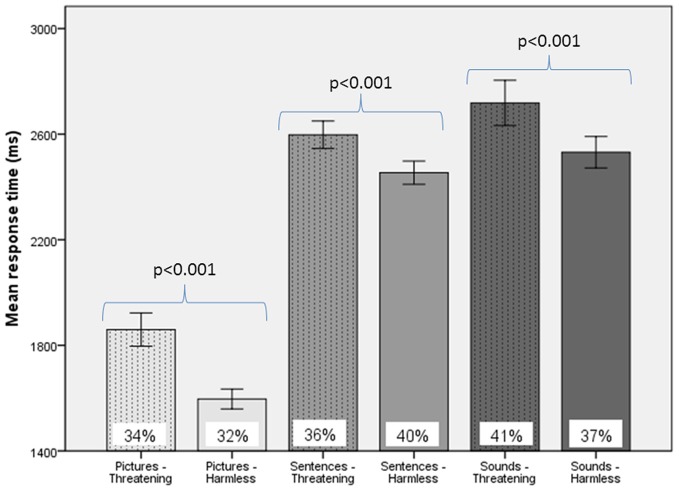
**Response times and frequency of evoked SCRs to picture, sentence, and sound stimuli.** “Threatening” responses are shown as dotted columns; “harmless” responses are shown as plain columns. Error bars are 95% confidence intervals. There was a main effect of modality on RTs [sounds longer RTs than sentences, which had longer RTs than pictures; *F*_(2, 48)_ = 98.05, *p* < 0.001], a main effect of subjective assessment on RTs [“threatening” longer RTs than “harmless”; *F*_(1, 24)_ = 14.51, *p* = 0.001], but no main effect of presence or absence of an SCR on RTs [*F*_(1, 24)_ = 0.26, *p* = 0.614; data not shown; repeated measures ANOVA]. There were no significant differences in the percentage of SCRs to stimuli assessed as “threatening” compared with those subjectively assessed as “harmless” (% figures in chart bars).

**Figure 3:**
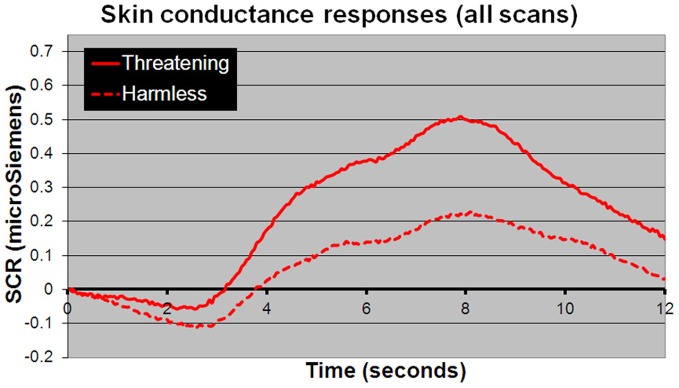
**Mean SCR amplitudes to all stimuli.** Stimuli subjectively assessed as “threatening” (solid line) compared with those assessed as “harmless” (dotted line) evoked significantly larger SCR amplitudes [*F*_(1, 24)_ = 8.32; *p* = 0.008]. The time course shown closely resembles a “typical” SCR, comprising an initial undershoot followed by a rise to peak 8 s after stimulus presentation returning to baseline within 12–14 s.

**Figure 4:**
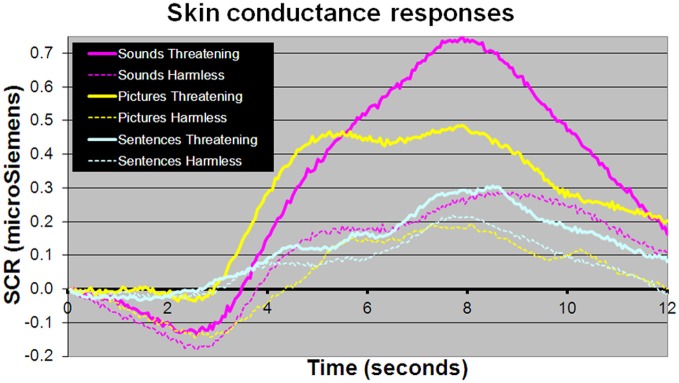
**Mean SCR amplitudes to picture, sentence, and sound stimuli**. Sound and picture stimuli subjectively assessed as “threatening” (solid lines) compared with those assessed as “harmless” (dotted line) evoked significantly larger SCR amplitudes (*t* = 2.65, *p* = 0.006 and *t* = 1.89, *p* = 0.033, respectively). There was no significant difference in SCR amplitudes between sentence stimuli subjectively rated as “threatening” or “harmless” (*p* > 0.1).

For behavioral RTs, in a 3 × 2 × 2 within-subject, repeated-measures ANOVA (picture or sentence or sound × “harmless” or “threatening” × presence or absence of an SCR), there was a main effect of subjective assessment [“threatening” longer RTs than “harmless”; *F*_(1, 24)_ = 14.51, *p* = 0.001; Figure 2], a main effect of modality [sounds longer RTs than sentences; sentences longer RTs than pictures; *F*_(2, 48)_ = 98.05, *p* < 0.001; Figure 2], but no main effect of the presence or absence of an SCR [*F*_(1, 24)_ = 0.26, *p* = 0.614]. There were no significant differences in the number of SCRs to stimuli assessed as “threatening” compared with those assessed as “harmless” (percentage figures in chart bars; Figure 2). However, for SCR amplitudes, a 3 × 2 within-subject, repeated-measures ANOVA (picture or sentence or sound × “harmless” or “threatening”), revealed a main effect of assessment [“threatening” greater SCR amplitude than “harmless”; *F*_(1, 24)_ = 8.32, *p* = 0.008; Figure 3] and a trend toward a main effect of modality [sounds greater SCR amplitudes than pictures; pictures greater SCR amplitudes than sentences; *F*_(2, 48)_ = 3.0, *p* = 0.059; Figure 4], but no interaction [*F*_(2, 48)_ = 2.22, *p* = 0.12]. *Post-hoc* pair-wise comparison (Tukey's HSD test) showed that “threatening” sounds and pictures were associated with significantly greater SCR amplitudes than sounds and pictures assessed as “harmless” (*t* = 2.65, *p* = 0.006 and *t* = 1.89, *p* = 0.033, respectively; Figure 4), but that there was no significant difference for sentences (*t* = 0.33, *p* = 0.372; Figure 4).

There was a significant positive correlation between an individual's average ISCR and number of stimuli assessed as “threatening” for sentences (*r* = 0.431, *p* = 0.016) and sounds (*r* = 0.385, *p* = 0.032), but not for pictures (*p* > 0.1). There were no significant correlations between ISCR or number of stimuli assessed as “threatening” and O-LIFE, EQ or PSQ scale scores (*p* > 0.1).

### fMRI—autonomic (ISCR) regressor

**Figure 5:**
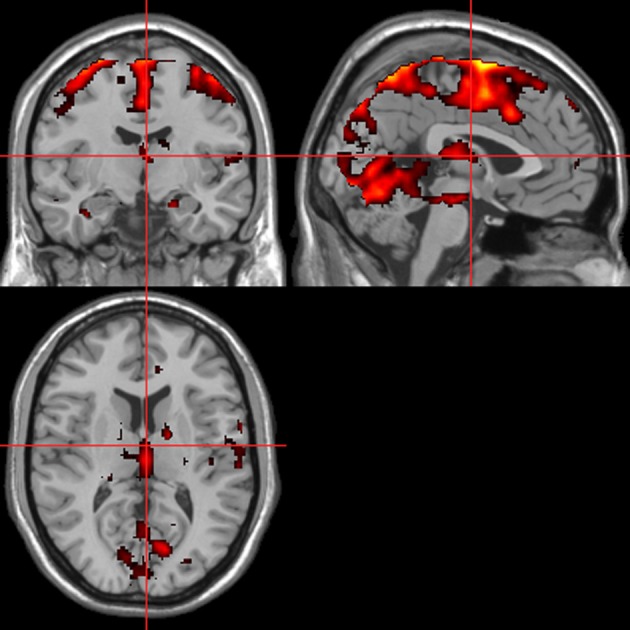
**ISCR regressor across picture, sentence, and sound stimuli.** Main effect of autonomic arousal. Flexible factorial design. *p* < 0.05 corrected for family-wise error (FWE). Extent threshold = 10. See Table 1 for anatomical descriptions and co-ordinates.

Across all stimuli (i.e., without differentiating between modalities), ISCR was associated with activations including bilateral precentral gyrus/supplementary motor area [SMA; Brodmann's Area (BA) 4/6], medial prefrontal cortex (mPFC; BA 8), precuneus/cuneus (BA 7/19), thalamus [dorso-medial (DM) nucleus], bilateral lingual gyrus (BA 18) and cerebellum (Table 1; Figure 5; *p* < 0.05 FWE). Separately, for picture, sentence and sound stimuli, this dorsal (precentral gyrus/SMA) and posterior (lingual gyrus/cerebellum) activation was replicated, though the DM-thalamic activation was only present for picture and sentence stimuli (i.e., not sounds). However, sound-stimuli ISCR data were associated with activation of left amygdala.

**Table 1:** **Picture, sentence, and sound stimuli. Brain activations associated with integrated skin conductance response (ISCR) activity (see Figure 5)**.

**Anatomical region**	**BA**	***x***	***y***	***z***	***Z*-value**	**Extent**
L postcentral gyrus	1/2/3	−30	−34	66	7.32	248
		*−38*	*−36*	*61*	*6.62*	
		*−48*	*−32*	*53*	*5.45*	
R postcentral gyrus	1/2/3	32	−36	64	5.84	45
L precentral gyrus	4	−38	−11	59	6.06	52
*L superior frontal gyrus*	*6*	*−28*	*−5*	*65*	*5.73*	
R precentral gyrus	4	44	−9	56	5.35	13
SMA/posterior mPFC	6	0	3	62	6.94	226
*R mid−cingulate gyrus*	*24*	*2*	*2*	*46*	*5.72*	
R SMA/MidFG	6	38	1	57	5.89	24
L mid−cingulate gyrus	24	−2	−11	43	5.18	15
R precuneus	7	6	−59	60	7.19	435
		*8*	*−41*	*68*	*6.62*	
		*6*	*−67*	*53*	*6.57*	
Lingual gyrus	18	−2	−87	−1	5.76	10
Cerebellum		4	−66	−8	5.71	75
*Lingual gyrus*	*18*	*2*	−*76*	−*10*	*5.00*	

*Co-ordinates are shown in standardized neuroanatomical space (Talairach and Tournoux, 1988). R, right; L, left; BA, Brodmann's area; SMA, supplementary motor area; mPFC, medial prefrontal cortex; MidFG, middle frontal gyrus; DM, dorso-medial. Co-ordinates without a corresponding extent threshold are shown in italics and refer to sub-clusters of the preceding activation. P < 0.05 corrected for family wise error (FWE)*.

### fMRI—behavioral response regressor

**Figure 6:**
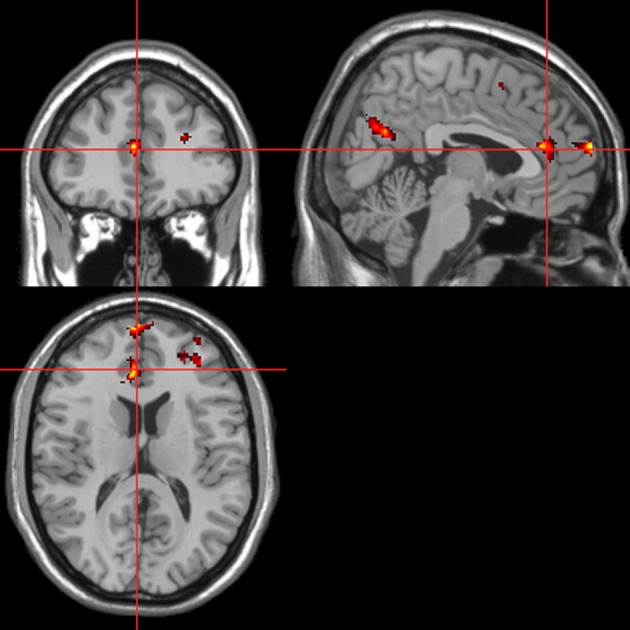
**“Threatening” > “harmless” regressor across picture, sentence, and sound stimuli.** Main effect of stimuli subjectively assessed as “threatening” across modalities. Flexible factorial design *p* < 0.001. Extent threshold = 10. See Table 2 for anatomical descriptions and co-ordinates.

**Figure 7:**
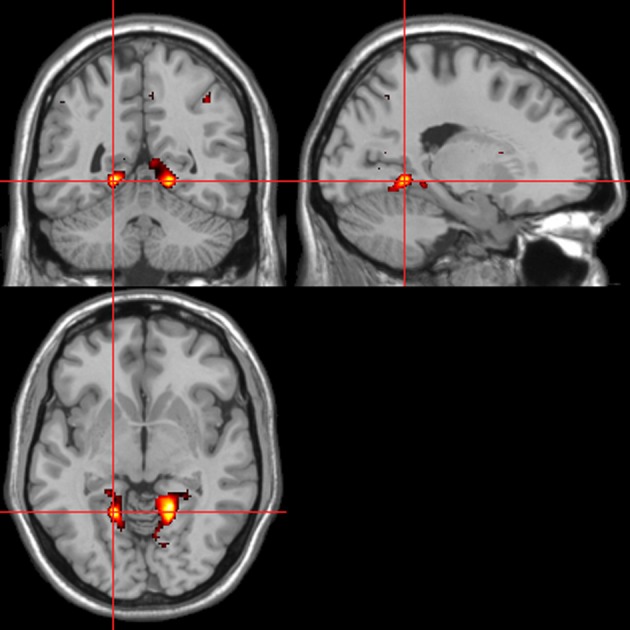
**Pictures.** Activations associated with “threatening” compared with “harmless” behavioral responses. Flexible factorial design *p* < 0.001. Extent threshold = 10. See Table 3A for anatomical descriptions and co-ordinates.

Across all stimuli, “threatening” compared with “harmless” behavioral assessments were associated with activation of bilateral middle frontal gyrus (MidFG; BA 10/46), mPFC/frontal pole (BA 10), anterior cingulate cortex (ACC; BA 24/32), precuneus (BA 7), and lingual gyrus (BA 18; Table 2; Figure 6). Threatening pictures were associated with activation including bilateral parahippocampal gyrus/lingual gyrus (BA 30/19), bilateral angular gyrus/temporo-parietal junction (BA 39), mPFC/ACC (BA 10/32), and posterior cingulate/precuneus (BA 31/7; Table 3A and Figure 7). This bilateral parahippocampal gyrus activation survived FWE correction at *p* < 0.05. Threatening sentences were associated with activation including bilateral MidFG/frontal pole (BA 10), bilateral ACC (BA 24), posterior cingulate and precuneus (BA 30/7; Table 4A and Figure 9). This left ACC activation survived FWE correction at *p* < 0.05. Threatening sounds were associated with activation including right transverse temporal gyrus (also known as Heschl's gyrus; BA 41) and bilateral middle/superior temporal gyrus (BA 21/22/42; Table 5A and Figure 11).

**Table 2:** **Pictures, sentences, and sounds. Brain activations associated with “Threatening” compared with “Harmless” behavioral judgments (see Figure 6)**.

**Anatomical region**	**BA**	***x***	***y***	***z***	***Z*-value**	**Extent**
L anterior cingulate cortex	24/32	−6	36	13	4.50	57
		*−8*	*43*	*14*	*3.49*	
L mPFC/frontal pole	10	−6	63	12	4.28	38
L middle frontal gyrus	10	−32	49	18	4.14	47
	*46*	*−24*	*53*	*18*	*3.50*	
R middle frontal gyrus	10	28	48	22	3.74	31
	10/46	36	41	11	3.24	11
Precuneus	7	−4	−61	27	3.88	90
	*31*	*4*	*−74*	*31*	*3.56*	
L precuneus	7	−16	−76	42	3.77	24
Lingual gyrus	18/19	2	−54	1	3.80	13

*Co-ordinates are shown in standardized neuroanatomical space (Talairach and Tournoux, 1988). R, right; L, left; BA, Brodmann's area; mPFC, medial prefrontal cortex. Co-ordinates without a corresponding extent threshold are shown in italics and refer to sub-clusters of the preceding activation. P < 0.001 uncorrected for multiple comparisons; extent threshold = 10*.

**Table 3A:** **Pictures. Brain activations associated with “Threatening” compared with “Harmless” behavioral judgments (see Figure 7)**.

**Anatomical region**	**BA**	***x***	***y***	***z***	***Z*-value**	**Extent**
R lingual/paraH gyrus	19/30	18	−51	−3	5.03	109
*R lingual gyrus*	*19*	*18*	*−59*	*−5*	*3.54*	
L lingual/paraH gyrus	19/30	−16	−47	−3	4.91	227
		*−8*	*−62*	*0*	*3.50*	
*L post. cingulate gyrus*	*23/31*	*−12*	*−56*	*12*	*3.50*	
Lingual gyrus	19/18	4	−58	1	3.63	16
R posterior insula/TTG	41	38	−17	16	4.49	45
L superior temporal gyrus	22	−50	−24	16	4.07	38
L MidFG/frontal pole	10	−34	51	16	4.00	21
L mPFC/ACC	10	−8	43	14	3.97	17
L anterior cingulate cortex	32	−14	34	19	3.87	20
		*−22*	*36*	*15*	*3.18*	
Anterior cingulate cortex	32/24	0	47	0	3.39	14
L inferior frontal gyrus	44/45	−34	11	16	3.85	16
mPFC/frontal pole	10	−4	65	12	3.85	11
R MidFG/frontal pole	10	40	47	12	3.63	15
L precuneus	7	−16	−76	42	4.42	78
L posterior cingulate	31	−12	−33	31	4.19	13
R precuneus	7	12	−72	40	3.76	44
		*14*	*−62*	*42*	*3.60*	
		*12*	*−68*	*48*	*3.35*	
Cuneus	17/31	0	−71	11	3.44	10
Precuneus/post. cingulate	31/23	−2	−63	25	3.37	41
	31/23	*−2*	*−68*	*33*	*3.35*	
R IPL/angular g./TPJ	40/39	40	−62	38	3.71	25
		*34*	*−68*	*42*	*3.33*	
L MTG/angular g./TPJ	39	−34	−65	29	3.71	22
L IPL/angular g./TPJ	40/39	−51	−60	40	3.65	17
R hippocampus/ParaH g.	30/19	24	−39	−6	3.65	26
L superior temporal gyrus	22/42	−61	−26	16	3.59	15

**Table 3B:** **Pictures. Brain activations associated with psycho-autonomic interaction (PAI) of integrated skin conductance response (ISCR) and behavioral response (see Figure 8)**.

**Anatomical region**	**BA**	***x***	***y***	***z***	***Z*-value**	**Extent**
Right fusiform gyrus	37	30	−40	−13	3.50	12
*Right fusiform gyrus/parahippocampal g*.	*37/19*	*36*	−*41*	−*8*	*3.40*	

*Co-ordinates are shown in standardized neuroanatomical space (Talairach and Tournoux, 1988). R, right; L, left; BA, Brodmann's area; paraH, parahippocampal; post., posterior; TTG, transverse temporal gyrus; MidFG, middle frontal gyrus; mPFC, medial prefrontal cortex; ACC, anterior cingulate cortex; IPL, inferior parietal lobule; TPJ, temporo-parietal junction; g., gyrus. Co-ordinates without a corresponding extent threshold are shown in italics and refer to sub-clusters of the preceding activation. P < 0.001 uncorrected for multiple comparisons; extent threshold = 10*.

**Table 4A:** **Sentences. Brain activations associated with “Threatening” compared with “Harmless” behavioral judgments (see Figure 9)**.

**Anatomical region**	**BA**	***x***	***y***	***z***	***Z*-value**	**Extent**
L anterior cingulate cortex	24	−10	15	23	5.01	190
		*−22*	*13*	*21*	*4.05*	
		*−22*	*3*	*24*	*4.01*	
R inferior frontal g./ACC	44/24	24	9	25	4.33	212
*R anterior cingulate cortex*		*12*	*15*	*21*	*4.14*	
*R* inferior frontal gyrus	*44*	*30*	*0*	*30*	*3.87*	
Medial prefrontal cortex	6/8	−2	12	51	3.67	12
L MidFG/frontal pole	10	−30	50	21	4.42	99
		*−26*	*55*	*17*	*3.59*	
R MidFG/frontal pole	10	24	51	20	4.29	90
		*32*	*45*	*16*	*3.86*	
		*24*	*45*	*14*	*3.79*	
L superior frontal gyrus	6	−18	13	58	*3.69*	12
R precuneus	7	8	−74	44	4.10	28
L cingulate gyrus	23	−8	−22	29	*3.98*	10
Precuneus	7	4	−54	56	3.96	51
		*−4*	*−59*	*56*	*3.45*	
Posterior cingulate gyrus	30/23	0	−50	10	3.35	10

**Table 4B:** **Sentences. Brain activations associated with psycho-autonomic interaction (PAI) of integrated skin conductance response (ISCR) and behavioral response (see Figure 10)**.

**Anatomical region**	**BA**	***x***	***y***	***z***	***Z*-value**	**Extent**
R putamen/ACC		22	19	−1	4.60	43
R putamen		18	8	0	3.53	11
R insula		34	8	1	3.90	102
R middle frontal gyrus	10	28	42	−9	3.73	12
L STG/MTG	22/21	−50	−14	−3	3.77	21
L thalamus (VPL n.)		−18	−15	4	3.71	20
L cerebellum		−24	−67	−20	4.05	28

*Co-ordinates are shown in standardized neuroanatomical space (Talairach and Tournoux, 1988). R, right; L, left; BA, Brodmann's area; ACC, anterior cingulate cortex; MidFG, middle frontal gyrus; STG, superior temporal gyrus; MTG, middle temporal gyrus; VPL n., ventro-postero-lateral nucleus. Co-ordinates without a corresponding extent threshold are shown in italics and refer to sub-clusters of the preceding activation. P < *0.001* uncorrected for multiple comparisons; extent threshold = 10*.

**Table 5A:** **Sounds. Brain activations associated with “Threatening” compared with “Harmless” behavioral judgments (see Figure 11)**.

**Anatomical region**	**BA**	***x***	***y***	***z***	***Z*-value**	**Extent**
R MTG/STG	22	51	−27	3	4.53	67
*R TTG (Heschl*'*s g.)*	*41*	*57*	*−25*	*10*	*3.74*	
R middle temporal gyrus	21	40	−41	−6	3.72	15
R middle temporal gyrus	21	51	−4	−10	3.70	40
R middle temporal gyrus	21	51	−54	5	3.60	11
L superior temporal gyrus	22/42	−40	−27	7	4.52	70
		*−44*	*−19*	*3*	*3.92*	
L MTG/STG	21/22	−55	−25	−2	4.29	25
L middle temporal gyrus	21	−55	−46	8	3.38	10
L MTG/STG	21	−53	−12	−3	3.33	11
R precentral gyrus	6	50	−6	32	4.00	16
R IPL/TPJ	40	38	−52	43	3.96	36
		*32*	*−58*	*40*	*3.48*	
R precentral gyrus	6	38	−10	32	3.96	42
L paraH/lingual gyrus	19	−18	−43	−3	3.54	14
L precuneus	31/7	−26	−45	34	3.50	24

**Table 5B:** **Sounds. Brain activations associated with psycho-autonomic interaction (PAI) of integrated skin conductance response (ISCR) and behavioral response (see Figure 12)**.

**Anatomical region**	**BA**	***x***	***y***	***z***	***Z*-value**	**Extent**
L middle cingulate gyrus	24	−6	−22	36	5.49	94
R anterior cingulate cortex	32	22	23	27	4.11	56
R anterior cingulate cortex	32	24	39	9	3.96	13
R ACC/IFG	32/44	30	11	29	3.77	11
L anterior cingulate cortex	32	−26	17	29	3.79	14
L anterior cingulate cortex	32	−12	11	29	3.74	6
R IPL/TPJ	40	48	−34	24	4.19	36
L superior frontal gyrus	10	−24	62	4	3.86	11
L inferior frontal gyrus	47	−38	33	−5	4.38	90
*L inferior frontal gyrus/MidFG/OFC*	*47/11*	*−34*	*34*	*−12*	*4.22*	
L inferior frontal gyrus	44	−42	7	25	3.64	24
R postcentral gyrus	1/2/3	48	−13	19	4.03	34
L postcentral gyrus	1 2 3	−32	−25	36	4.41	39
*L postcentral gyrus/precentral gyrus*		*−36*	*−18*	*30*	*3.45*	
L precentral gyrus	4	−12	−20	65	3.63	11
L SMA/precentral gyrus	6/4	−55	0	33	3.62	46
		*−48*	*−2*	*31*	*3.62*	
		*−48*	*−12*	*36*	*3.34*	
R precuneus	7	22	−56	47	3.66	12
L hippocampus		−40	−20	−9	3.84	10

*Co-ordinates are shown in standardized neuroanatomical space (Talairach and Tournoux, 1988). R, right; L, left; BA, Brodmann's area; MTG, middle temporal gyrus; STG, superior temporal gyrus; TTG, transverse temporal gyrus (also known as Heschl's gyrus); IPL, inferior parietal lobule; TPJ, temporo-parietal junction; paraH, parahippocampal; ACC, anterior cingulate cortex; IFG, inferior frontal gyrus; MidFG, middle frontal gyrus; OFC, orbitofrontal cortex; SMA, supplementary motor area. Co-ordinates without a corresponding extent threshold are shown in italics and refer to sub-clusters of the preceding activation. P < 0.001 uncorrected for multiple comparisons; extent threshold = 10*.

### fMRI—autonomic (ISCR)-behavioral response convolved regressor

**Figure 8:**
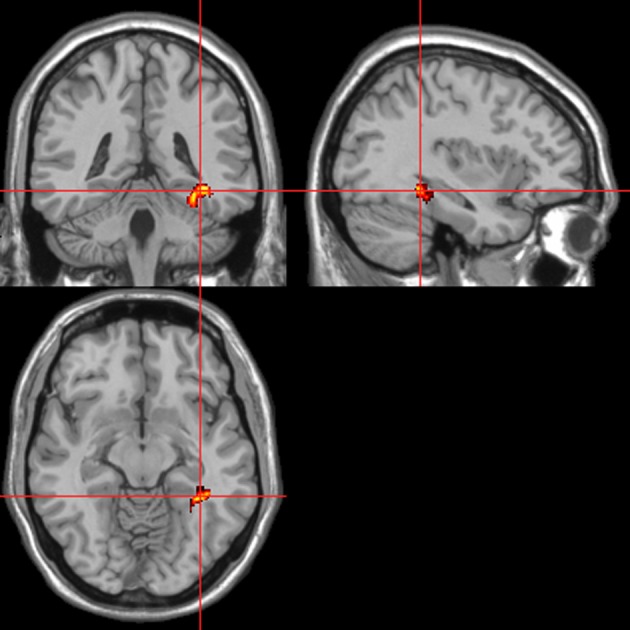
**Pictures.** Psycho-autonomic interaction (PAI) between integrated skin conductance response (ISCR) and behavioral response (“threatening” > “harmless”). Flexible factorial design *p* < 0.001. Extent threshold = 10. See Table 3B anatomical descriptions and co-ordinates.

Across all stimuli, the interaction between autonomic (ISCR) and “threatening” or “harmless” assessment responses—our PAI was associated with activation of right MidFG (BA 10; T&T co-ordinates 24 42 −7) and left mid-cingulate gyrus (BA 24; −6 −23 36). Threatening picture-ISCR interactions were associated with activation of right fusiform gyrus/parahippocampal gyrus (BA 37; Table 3B and Figure 8). Threatening sentence-ISCR interactions were associated with activation of right insula and MidFG (BA 10), left thalamus [ventral posterolateral (VPL) nucleus], left superior temporal gyrus (BA 22) and left cerebellum (Table 4B and Figure 10). Threatening sound-ISCR interactions were associated with activations including left mid-cingulate gyrus, bilateral postcentral gyrus (BA 1/2/3), bilateral IFG (BA 44/47) and right inferior parietal lobule (BA 40; Table 5B and Figure 12). This left mid-cingulate gyrus activation survived FWE correction at *p* < 0.05.

**Figure 9:**
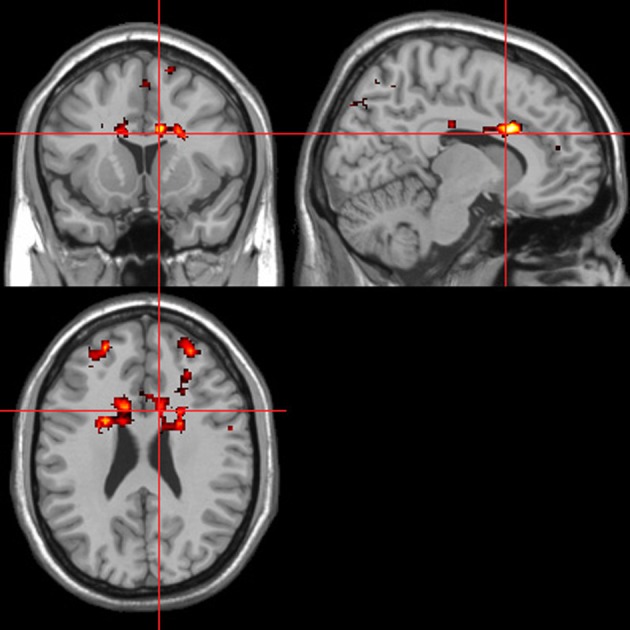
**Sentences.** Activations associated with “threatening” compared with “harmless” behavioral responses. Flexible factorial design *p* < 0.001. Extent threshold = 10. See Table 4A for anatomical descriptions and co-ordinates.

**Figure 10:**
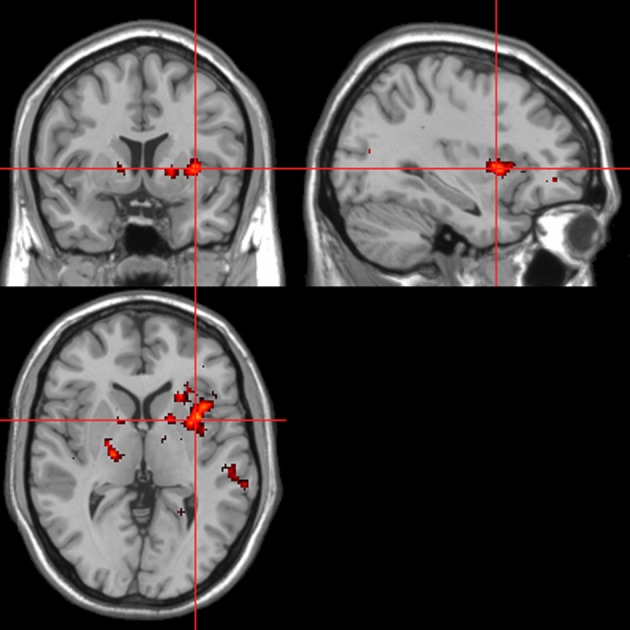
**Sentences.** Psycho-autonomic interaction (PAI) between integrated skin conductance response (ISCR) and behavioral response (“threatening” > “harmless”). Flexible factorial design *p* < 0.001. Extent threshold = 10. See Table 4B for anatomical descriptions and co-ordinates.

**Figure 11:**
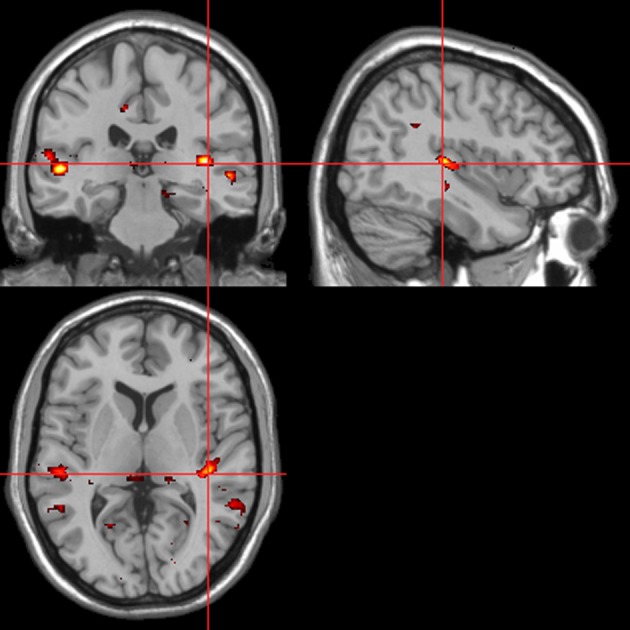
**Sounds.** Activations associated with “threatening” compared with “harmless” behavioral responses. Flexible factorial design *p* < 0.001. Extent threshold = 10. See Table 5A for anatomical descriptions and co-ordinates.

**Figure 12:**
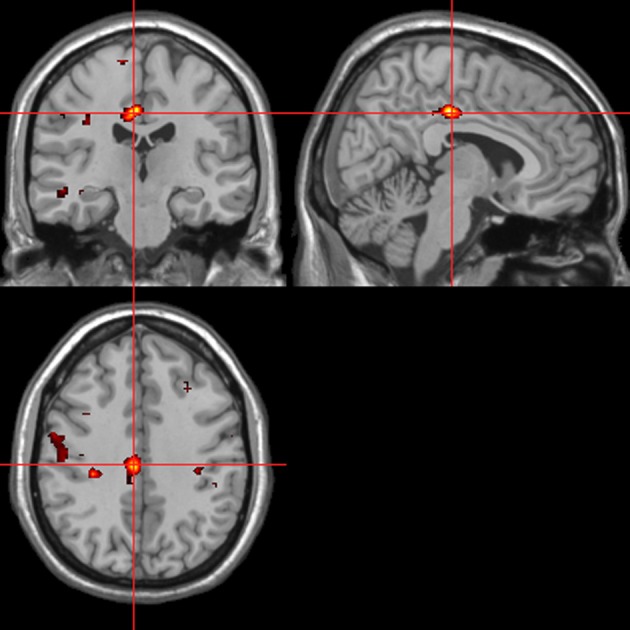
**Sounds.** Psycho-autonomic interaction (PAI) between integrated skin conductance response (ISCR) and behavioral response (“threatening” > “harmless”). Flexible factorial design *p* < 0.001. Extent threshold = 10. See Table 5B for anatomical descriptions and co-ordinates.

## Discussion

In agreement with our first and second hypotheses, picture, sentence, and sound stimuli subjectively assessed as “threatening” compared with those assessed as “harmless” had significantly longer RTs and increased SCR amplitudes (except non-significantly for sentence SCR amplitudes). Parieto-occipito-thalamic brain regions were associated with autonomic arousal (ISCR) across stimulus modalities, in broad agreement with previous research (Fredrikson et al., 1998; Patterson et al., 2002). Across stimulus modalities, stimuli assessed as “threatening” activated prefrontal and precuneus regions, but in contrast to the ISCR findings, there were very clear modality-specific activations. The ISCR-behavioral response convolution (PAI) analysis revealed modality-specific activations which were distinct from those seen in the separate ISCR and behavioral-response analyses. Subjects' average ISCRs were positively correlated with the number of sentence and sound stimuli assessed as “threatening.” Contrary to our remaining hypotheses we did not find that stimuli assessed as “threatening” were routinely associated with supra-threshold amygdala activity or a relationship between schizotypal personality traits and autonomic or behavioral responses.

The brain areas associated with autonomic arousal, which function in parallel with cognitive assessment of environmental stimuli, included left amygdala (sounds), dorsomedial thalamic nucleus (pictures and sentences), precuneus, lingual gyrus, and motor cortex (bilateral precentral gyrus/SMA). The amygdala, thalamic, precuneus, and SMA activations are likely directly related to autonomic arousal (Critchley et al., 2003; Napadow et al., 2008; Zhang et al., 2012). The lingual gyrus has previously been associated with the generation and representation of SCRs (Critchley et al., 2000). The precuneus has also been associated with emotional self-regulation (Johnston et al., 2010) whilst the motor cortex has been associated with internal attributions of events whether or not the “self” was viewed as an active intentional agent (Blackwood et al., 2000). Activation of the motor cortices may also prepare the body to move away from threat, though some research has actually reported a decreased activity in primary motor cortex during anticipation of an aversive event (cognitively induced fear; Butler et al., 2007). However, it has also been reported that different aspects of the emotional response, namely arousal and valence, may be mediated by different brain circuits (Anders et al., 2004). Anders and colleagues, using human and animal pictures from the IAPS, studied the trial-by-trial correlation of brain activation with SCR, startle response and subjective ratings of valence and arousal. Post-scan arousal reports to individual pictures were positively correlated with SCR. Using a region-of-interest approach Anders and colleagues showed that activation of the amygdala and insula positively correlated with valence ratings, whilst arousal ratings were correlated with thalamic and frontomedial cortex activity. Peripheral physiologic responses (SCR and startle response) were localized to regions of anterior parietal cortex, primarily somatosensory association areas. Furthermore, Anders and colleagues report a functional segregation of brain structures differentiating SCR and startle responses from verbal responses. Specifically, whilst SCRs were associated with frontomedial cortex activity and startle responses with amygdala activity, verbal ratings of valence and arousal were associated with activation of insula and thalamus respectively.

Contrary to our third hypothesis, we did not find amygdala activation to be routinely associated with all stimuli assessed to be “threatening.” One possible explanation for this lack of robust amygdala activation is that rather than being necessary for fear perception *per se*, the amygdala is active when the rest of the brain cannot easily predict (1) what sensations mean? (2) what to do about them? or (3) what value they hold in that context? (Lindquist et al., 2012). Hence, the subjectively variable level of threat conferred by our stimuli may have lessened the difference in amygdala activation between “harmless” and “threatening” subjective assessments. An alternative explanation is that if a proportion of stimuli subjectively assessed as “harmless” were actually experienced as pleasant and thus led to positive arousal and hence amygdala activation, that our main contrast of interest (i.e., threatening > harmless), would not have shown a significant difference in relative amygdala activation.

Our modality-specific hypotheses of areas more activated by “threatening” than “harmless” assessments were in the main confirmed for pictures (lingual gyrus, parahippocampal gyrus, and mPFC) and sounds (right Heschl's gyrus and bilateral superior temporal gyrus), but less so for sentences, where the bilateral dorsal (cognitive) ACC and MidFG activations were predominant (as opposed to the left IFG which we hypothesized). One possible explanation for the lack of predicted activations to threatening sentences is that our hypothesis was based on previous research into threatening versus non-threatening words (Isenberg et al., 1999; Blackwood et al., 2000; Compton et al., 2003) which may require less cognitive processing and deliberation than full sentences. Sensory facilitation of auditory cortex by emotional cues as we have shown was recently reported (Plichta et al., 2011) in a functional near-infrared spectroscopy (fNIRS) study using pleasant, unpleasant and neutral sounds from the International Affective Digitized Sound System (IADS; Bradley and Lang, 1999) database. However, Plichta and colleagues report that both pleasant and unpleasant sounds led to significantly greater auditory cortex activation than neutral sounds, with no significant difference between pleasant and unpleasant. As our present study involved subjects making assessments on a “harmless”–“threatening” binary dimension, it is likely that our “harmless” category contained stimuli which could be described as both “pleasant” and “neutral”.

The dorsal (cognitive) division of ACC which was activated by threatening pictures and sentences, is classically associated with error detection and monitoring as opposed to the ventral (affective) ACC which is classically associated with assessing the salience of emotional information (Bush et al., 2000). Though by this “classical model,” activation of ventral ACC would better fit with the task demands, recent research (Shackman et al., 2011) has argued for a more general role for the anterior midcingulate cortex (aMCC), specifically in generating aversively motivated behavior across affect, pain and cognition. This “adaptive control hypothesis” by which the aMCC activates when the most adaptive course of action is uncertain and outputs to motor centers executing goal-directed behavior fits neatly with making subjective assessments of potentially threatening environmental stimuli. An alternative explanation for the brain activations seen for the threatening-harmless contrasts is that they reflect the fronto-parietal networks implicated in top-down attention (Corbetta and Shulman, 2002) and that threatening stimuli elicited more attention than harmless ones. This latter explanation and the “adaptive control hypothesis” are of course not mutually exclusive.

Our ISCR-behavioral response convolution (PAI) analyses were designed to reveal brain regions above and beyond those BOLD activations attributable to autonomic and behavioral responses separately. Results included right parahippocampal gyrus for pictures, right insula and ACC for sentences and left mid-cingulate gyrus/bilateral IFG for sounds. It is noteworthy from these modality-specific findings that there was greater interaction between SCR and behavior in high order visual cortex (Malach et al., 2002) for pictures and that the role of the insula in the detection and awareness of bodily changes (“interoception”) has been the subject of much recent research (Craig, 2003, 2009; Critchley et al., 2004; Simmons et al., 2006; Singer et al., 2009) as these bodily changes may modulate cognitive interpretation and hence behavior. As regards the activations obtained by the PAI for sounds, previous research into the SCR orienting response (Williams et al., 2000) reported that “significant” compared with “familiar” stimuli activated brain regions including ventral ACC and ventral mPFC.

As we had no implicit baseline, our main contrast of interest compared how subjects assessed the subjective valence of stimuli. Hence our power to detect significant differences between conditions was restricted by the relatively subtle difference between the “active” and “baseline” conditions (stimuli assessed as being “threatening” and “harmless,” respectively) and relevant analyses are reported at an uncorrected statistical threshold. Such a liberal threshold is in line with recent guidelines for analysis of complex social neuroscience paradigms (Lieberman and Cunningham, 2009). Similarly, the minimum extent threshold chosen (10 voxels) for the novel imaging contrasts was justified in our original ethics and research protocol as appropriate due to the exploratory nature of study.

Whilst a large proportion of the reported results are in line with our original *a priori* hypotheses, they are also occasionally at odds with more recently reported results of the neural and autonomic correlates of affective processing (i.e., those published after the present study was begun). Critchley (2009) in a review of the extant literature highlights the role of the anterior cingulate and insula in the response and representation of bodily states in specific behavioral contexts. Though we reported activation of right insula associated with autonomic arousal, our activation of anterior cingulate was primarily associated with “threatening” behavioral assessments. However, another recent study (Zhang et al., 2012), using a stop signal task to examine the neural correlates of SCRs reported activation of the SMA, middle cingulate gyrus and precuneus, findings which are much more in agreement with the current findings. Another recent study (Henderson et al., 2012) measured the neural correlates of spontaneous fluctuations in skin sympathetic nerve activity (SSNA) via direct recording from the common fibular nerve (as opposed to inferring SSNA from SCR). Using positively and negatively charged emotional images from the IAPS dataset to evoke autonomic arousal, SSNA was associated with more frontal regions (including orbital, dorsolateral, and vmPFC) than has generally been previously reported. Henderson and colleagues did however also report robust activation of right precuneus as we have done in the present study. Finally, two recent studies have examined the role of personality in modulating neural responses to anticipating threat in the form of electric shocks (Drabant et al., 2011) and neural and autonomic responses to threatening facial expressions and body postures (Kret et al., 2011). In Drabant and colleagues' study, shock anticipation was associated with increased SCRs and corresponding activation of brain areas, many of which overlap with those reported in the present study, including precentral gyrus, thalamus, insula, and mid-cingulate cortex (ACC). Individual neuroticism scores in Drabant and colleagues' study were negatively correlated with activation of left IFG and insula. Kret and colleagues meanwhile examined the influence of negative affectivity and social inhibition on neural responses to videos of fearful and angry actors. While individuals with increased negative affectivity showed reduced activation of core emotion systems (including cortical and sub-cortical regions such as amygdala) socially inhibited individuals over-activated a broader, though exclusively cortical, network (including temporo-parietal junction, superior temporal gyrus, and orbitofrontal cortex).

Contrary to our final hypothesis we did not find a relationship between personality traits (as measured by the O-LIFE, EQ, and PSQ) and behavioral or autonomic responses. Previous studies have suggested a relationship between the main personality dimensions (the so-called “big five”; Digman, 1990) and SCR latency, but not magnitude (Mardaga et al., 2006). Our finding of a positive correlation between an individual subject's average ISCR and the number of “threatening” assessments they made suggests that such behavioral-autonomic modulations may be present over state- or mood-length periods, but are not related to measures of sub-clinical psychosis-proneness. This correlation between ISCR and “threatening” responses may also be related to an individual's underlying neuroticism (Drabant et al., 2011), a personality trait which we did not directly measure.

## Limitations

We have utilized a relatively liberal height and extent threshold for our fMRI results, which may have led to reporting of some Type I errors (i.e., false positives). However, use of a mapwide false discovery rate (FDR) and family-wise error (FWE) of *p* < 0.05 has been reported to be unduly conservative for novel complex cognitive and affective social neuroscience processes as were examined in this study (Lieberman and Cunningham, 2009). Use of the “sparse” fMRI sequence was required for delivery of sound stimuli and was kept for picture and sentence stimuli to facilitate inter-modality comparison. However, this necessarily restricted the time sampling window, though the data collection period was targeted at a period immediately after task completion, utilizing the physiological delay and dispersion between neuronal activity and its resulting hemodynamic response (Eden et al., 1999). The lack of an implicit baseline condition was considered a worthwhile trade-off to obtain greater statistical power for the relatively subtle main contrast of interest (i.e., “threatening” > “harmless”). However, this prevented us from examining the main effect of “harmless” + “threatening” assessments to offer evidence regarding current speculations on the amygdala being a novelty detector, rather than a threat detector (Blackford et al., 2010). Our use of only male volunteers also means that we are unable to comment on the possible gender-specific nature of any activations or behavioral response characteristics.

Finally, our hypotheses in this initial study were restricted to greater brain activations to “threatening” compared with “harmless” stimuli, and brain activations positively correlated with ISCR. Consequently we had no specific *a priori* hypotheses about, and so insufficient power to confidently interpret, activations related to the reverse contrasts (“harmless” greater than “threatening” or negative correlations with ISCR).

## Future studies

Whilst the SCR may provide a purer measure of sympathetic activity than heart rate or pupil diameter (Wallin, 1981; Öhman et al., 2000), a future study may benefit from examining more than one of these, as there is evidence that SCR and heart rate may separately code the arousal and valence aspects of affective experience, respectively (Bradley et al., 2001). Future studies may also benefit from a measure of an individual's sensitivity to visceral cues such as heartbeat-detection (Katkin et al., 2001). Katkin and colleagues used backward-masked images of fear-relevant stimuli to show that subjects who could detect their heartbeats performed better than chance at predicting a forthcoming electric shock associated with the conditioned stimuli. Hence, a measure of interoceptive sensitivity to sympathetic arousal could index an underlying trait-bias toward negative interpretations of “ambiguous” stimuli (Richards et al., 2003). These hunches or “gut feelings” may be another important modulator of cognitive evaluation of emotionally salient stimuli (Dalton et al., 2005), and hence important in our understanding of the role of relevant structures such as the insula. Investigation of the strength of this negativity-bias may also benefit from a continuous rating scale of “threat” as opposed to a binary forced-choice metric. Such a continuous rating scale may also be beneficial in separating genuinely “threatening” stimuli from more generally “negative” stimuli, which may have been classified as “threatening” when given a binary choice and have therefore contributed little signal, but potentially problematic noise to the relevant fMRI contrasts.

## Conclusions

In summary, convolving concurrently acquired SCR and fMRI measurements during assessment of potentially threatening stimuli allows more sophisticated assessment of the component processes which comprise an “emotional response.” Our data are broadly, but not fully in line with previous studies. Hence, further studies are likely required to provide a baseline against which to test future hypotheses about cognitive and autonomic system interaction abnormalities which may underlie various neuropsychiatric disorders.

### Conflict of interest statement

The authors declare that the research was conducted in the absence of any commercial or financial relationships that could be construed as a potential conflict of interest.

## Appendix

**Table A1:** **Stimuli used (Available as online supplementary material)**.

**No**	**Sound**	**Sentence**	**Picture (IAPS^a^ code)**
1	Aeroplane	A car full of friends pulled up beside me	Snake (1050)
2	Alligator	A group of people followed me into a lift	Snakes (1111)
3	Ambulance	A stranger followed me down a dark alley	Spider (1201)
4	Battle	The shopkeeper took a photo of me	Pit bull (1300)
5	Bleeper	A child in the park smiled at me	Dog (1303)
6	Blender	The intruder turned my phone off	Women (1340)
7	Car crash	My mother walked into my house	Giraffes (1601)
8	Cello	A carpenter with a chainsaw looked at me	Mickey (1999)
9	Chains	People glared at me and left as I walked in	Woman (2030)
10	Clock	A stranger put a tablet in my drink	Woman (2037)
11	Cougar	A neighbor followed me down a dark street	Neu woman (2038)
12	Cow	A lady with shopping stood behind me	Clowns (2092)
13	Creaky floor	A group of angry boys chased me to my car	Family (2154)
14	Didgeridoo	A friend waved at me as I passed by	Farmer (2191)
15	Donkey	The gas man stormed into my house	Fingerprint (2206)
16	Drill	An unknown man stood in my front garden	Judge (2221)
17	Earthquake	A gang of teenagers watched me at the cash point	Butcher (2235)
18	Electric	People stopped talking when I entered the cafe	Lonely boy (2272)
19	Factory	A friend followed me into my garden	Family (2299)
20	Fireworks	A woman asked which house I lived in	Girl (2320)
21	Flicker	A builder with a cement mixer whistled at me	Father (2339)
22	Footsteps 1	My sister offered to hold my drink	Woman (2375.1)
23	Footsteps 2	My father threw a book at me	Boy (2391)
24	Forest	A girl screamed when she saw me	Medical worker (2394)
25	Gate closing	A group of teenagers shouted at me	Boy (2410)
26	Gorilla	A colleague turned the TV off	Elderly man (2520)
27	Grind	A child threw their rattle at me	Picnic (2560)
28	Growl	A group of girls followed me into a shop	Dance (2605)
29	Gunfire	My best friend chased me with a dagger	Police (2682)
30	Hacksaw	My boss threw his car keys at me	War (2683)
31	Hairdryer	A gang of teenagers gave me flowers	Shopping (2745.1)
32	Harmonica	My friend followed me with a sandwich	Drunk driving (2751)
33	Harp	Someone behind me shouted my name	Shadow (2880)
34	Helicopter	A masked man pointed his finger at me	Erotic female (4002)
35	Jungle	An angry man with an axe came toward me	Prostitute (4233)
36	Keys	An old man on the train stared at me	Attractive man (4532)
37	Modem	A girl across the street shouted my name	Couple (4598)
38	Monkey	A famous pop star waved at me	Wedding (4626)
39	Blackboard	A stranger watched me leave my house	Pine needles (5120)
40	Owl	A woman spat at me in the street	Nature (5220)
41	Race car	A man offered to buy me a drink	Mountains (5628)
42	Rain	A child watched me eating chocolate	Winter street (5635)
43	Ripping	A boy aimed a gun at my head	Prison (6000)
44	Roadworks	My friend turned off all the lights	Electric chair (6020)
45	Screech	A man with a baseball bat asked me for money	Ice cream (6250.2)
46	Shovel	A hairdresser cut all my hair off	Abduction (6312)
47	Sleigh bells	A girl dressed all in black stared at me	Attack (6561)
48	Snare	A soldier threw a grenade at me	Meat slicer (7361)
49	Stream	A man in sunglasses followed me to the shop	Store (7495)
50	Submarine	Some people looked up as I entered a cafe	Castle (7502)
51	Telephone	A man with a gun stood behind me at the bus stop	Jet (7620)
52	Thunder	A friend asked for my phone number	Motorcycle (8251)
53	Tornado	A car full of strangers followed me in the dark	Roller coaster (8490)
54	Tractor	A famous footballer swore loudly at me	Roller coaster (8499)
55	Vacuum	A stranger forced his way into my house	Scared child (9041)
56	Wasp	A nurse pointed a needle at me	Pollution (9341)
57	Waterfall	A young lady came into my house with a knife	Ticket (9417)
58	Whale	A stranger smiled at me in the street	Assault (9429)
59	Wind	An unknown car drove past me several times	Dental exam (9584)
60	Wolf	A boy with a cigarette stood behind me	KKK rally (9810)

a *Lang et al., 1997*.
